# Correction: Role of annealing temperature on the sol–gel synthesis of VO_2_ nanowires with *in situ* characterization of their metal–insulator transition

**DOI:** 10.1039/c8ra90076k

**Published:** 2018-09-26

**Authors:** Y.-R. Jo, S.-H. Myeong, B.-J. Kim

**Affiliations:** School of Materials Science and Engineering, Gwangju Institute of Science and Technology (GIST) 123 Cheomdangwagi-ro, Buk-gu Gwangju Korea kimbj@gist.ac.kr +82-062-715-2341

## Abstract

Correction for ‘Role of annealing temperature on the sol–gel synthesis of VO_2_ nanowires with *in situ* characterization of their metal–insulator transition’ by Y.-R. Jo *et al.*, *RSC Adv.*, 2018, **8**, 5158–5165.

The acknowledgements section in the published article omitted a funder and should instead read: This work was supported by the Samsung Research Funding Center of Samsung Electronics under Project Number SRFC-MA1402-10 and the National Research Foundation of Korea (NRF) under Grant NRF2013S1A2A2035468.

The Royal Society of Chemistry apologises for these errors and any consequent inconvenience to authors and readers.

## Supplementary Material

